# Analysis of mutations in Chinese patients with polycystic kidney disease by targeted exome sequencing

**DOI:** 10.1016/j.gendis.2024.101246

**Published:** 2024-02-28

**Authors:** Kaili Qin, Qian Wang, Jianbo Qing, Yaheng Li, Hao Gong, Zhijian Zha, Bingrui Zhou, Yafeng Li

**Affiliations:** a Department of Nephrology, Shanxi Provincial People’s Hospital (Fifth Hospital) of Shanxi Medical University, Taiyuan 030012, China; b Shanxi Provincial Key Laboratory of Kidney Disease, Shanxi Provincial People’s Hospital, Taiyuan 030012, China; c Department of Biochemistry & Molecular Biology, Shanxi Medical University, Taiyuan 030001, Shanxi, China; d Third Clinical School, Shanxi University of Traditional Chinese Medicine, No. 89, Sec. 1, Jinzi Road, Wanbailin District, Taiyuan City, Shanxi Province, China; e Shanxi Key Laboratory of Birth Defect and Cell Regeneration, Shanxi Medical University, Taiyuan, 030001, Shanxi, China; f Department of Nephrology, Hejin Municipal People’s Hospital, Yuncheng 043300, China; g Core Laboratory, Shanxi Provincial People’s Hospital (Fifth Hospital) of Shanxi Medical University, Taiyuan 030012, China; h Academy of Microbial Ecology, Shanxi Medical University, Taiyuan 030012, China

In recent years, molecular diagnostics has become pivotal in the detection of polycystic kidney disease (PKD).^1^ Nevertheless, given the extensive genomic architecture, allelic heterogeneity, and dispersed mutations in affiliated genes, the translation of its clinical prospects is constrained.^2^ Moreover, summarizing the pertinent literature within the past decade reveals that the majority of studies predominantly focus on three main genes (*PKD1*, *PKD2*, and *PKHD1*), resulting in a limited scope of genes considered (Table S1). In the present investigation, endeavoring to supersede the limitations of traditional genetic diagnosis methods, we employed next-generation sequencing to meticulously interrogate the entire coding domains and exon-intron junctions of 15 pre-eminent genes (Table S2).

In the study, 95 participants were recruited from Shanxi Provincial People's Hospital. Of the enrolled participants, 83 patients were diagnosed with PKD, 10 were suspected of PKD, and 2 participants had no confirmed PKD (Table S3). After assessing the participants' fundamental conditions, age, gender, and pertinent clinical data were compiled. In addition, the glomerular filtration rate was determined utilizing the CKD-EPI equation to assess the chronic kidney disease stage (Table S4).^3^ Subsequently, 95 volunteers underwent next-generation sequencing to detect the full coding region and exon-intron junction region associated with PKD (Table S5).

In this cohort, the age range of the participants varied from 5 to 85 years, with an average age of approximately 50 years (males: mean = 52.55 ± 14.36; females: mean = 48.54 ± 17.40). By analyzing the sequencing outcomes, 82 mutation sites were identified, predominantly in the *PKD1* (43%), *PKD2* (15%), and *PKHD1* (13%) (Fig. 1A). Statistics were performed on all detected mutation sites, most of which were missense mutations (60%), followed by frameshift mutations, accounting for 19%; the rest were nonsense mutations (8%), in-frame mutations (4%), and splice site mutations (7%) (Fig. 1B and Table S6). The pathogenicity of these mutation sites was analyzed, with the statistical results elucidating that the majority of them represented variants of uncertain significance (48/82), followed by pathogenic and likely pathogenic variants (28/82). The remaining minority consisted of benign and likely benign variants (3/82) (Fig. 1C, D and Table S7). The distribution of *PKD1*, *PKD2*, and *PKHD1* on exons and the number of various variant types were displayed in Figure 1E, and it could be seen that *PKD1* exon 15 was the one with the highest number of pathogenic variants, but this result can be attributed to its large genomic size (3620 bp).

**Figure 1:**
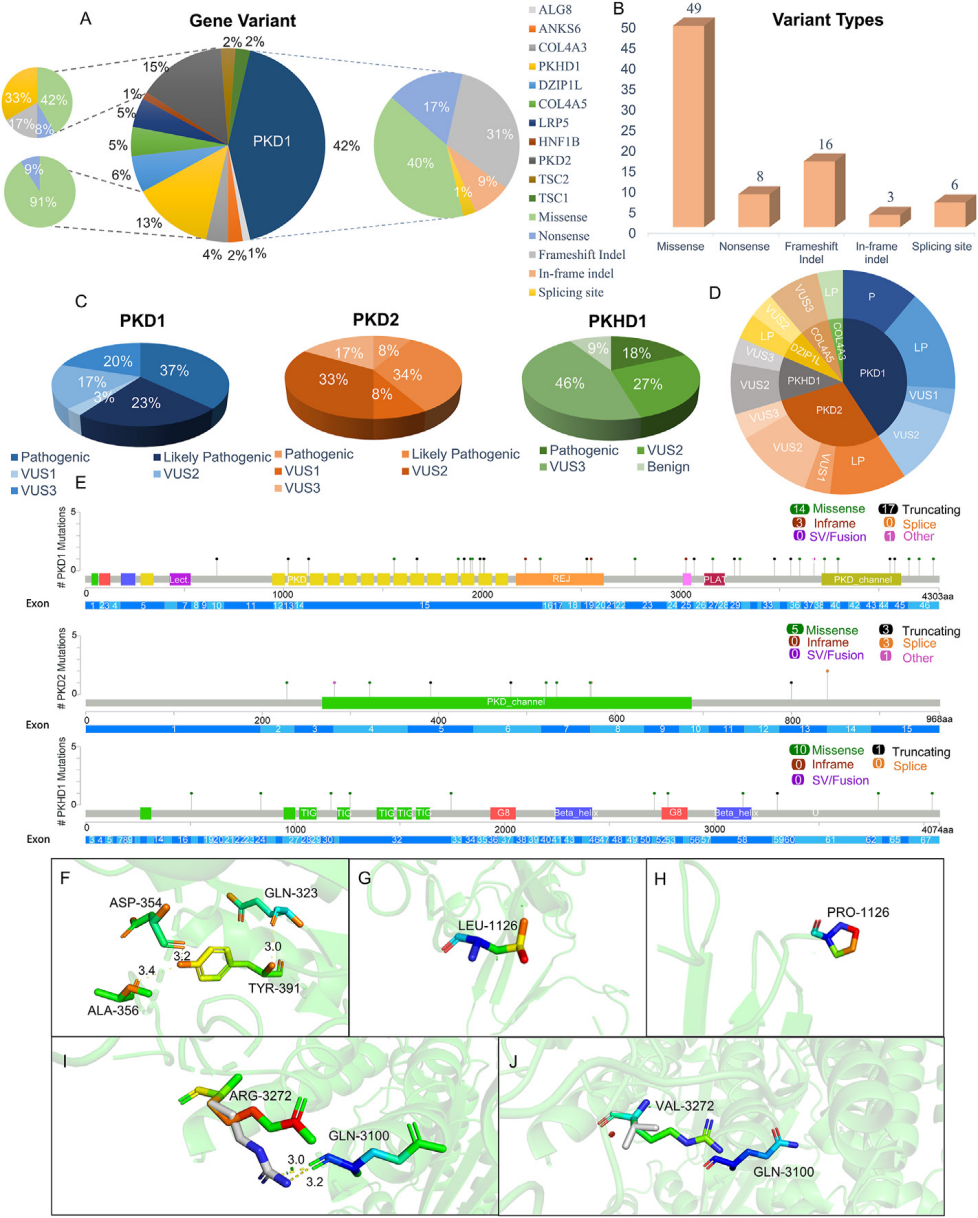
Mutation profiles of 95 participants. **(A)** Results of genetic sequencing of participants and types of variants in *PKD1*, *PKD2*, and *PKD3*. **(B)** Statistics of variant types. **(C)** Pathogenic classification of mutation sites. Pathogenicity is divided into pathogenic, likely pathogenic, variant of uncertain significance 1, variant of uncertain significance 2, variant of uncertain significance 3, likely benign, and benign. **(D)** Novel variant pathogenic classifications and proportions. **(E)** Variants distribution of *PKD1*, *PKD2*, and *PKHD1*. Figures were created and modified using the MutationMapper. **(F)** Relationship between amino acid at position 391 in the *PKD2* and adjacent amino acids. **(G)** The unmutated structure of the 1126-position amino acid in the *PKD1*. **(H)** Structure of amino acid mutations at position 1126 in the *PKD1*. **(I)** The relationship between amino acids at position 3272 and amino acids at position 3100 in the *PKD1* when they are not mutated. **(J)** Structure of amino acid mutation at position 3272 in the *PKD1*. In figures F and I, the yellow dotted lines represent hydrogen bonds, and 3.0, 3.2, and 3.4 represent the length of hydrogen bonds.

Remarkably, 27 novel mutation sites were identified, including 11 novel sites in *PKD1*, 8 in *PKD2*, 3 in *PKHD1*, 2 in *DZIP1L*, 1 in *COL4A3*, and 2 in *COL4A5*. After analyzing the sequencing results of the P51 patients, a novel missense mutation at the exon 5 region of the *PKD2*: c.1173T > G:p.Tyr391Ter, combined with a known missense mutation in *ANKS6* and *TSC1*. Hydrogen bonds can be formed between the tyrosine at position 391 of PC2 and the amino acids at positions 323, 354, and 356 in the PC1 protein. Nonetheless, the transition of thymidine nucleotide to guanine nucleotide at the 1173 site results in the conversion of tyrosine to a stop codon, disrupting the hydrogen bond and thereby reducing the interaction between PC1 and PC2, leading to alterations in protein structure and function (Fig. 1F).

In the analysis of the genetic test results of P77 patients, it was found that *PKD1*, *PKHD1*, and *DZIP1L* were mutated, of which the mutation of gene *PKD1* was novel, and the mutation of the remaining two genes has been described. A duplication of a gene locus was found in exon 15 of the gene *PKD1*: c.3376dup: p.Leu1126ProfsTer10. The structural changes of the 1126-bit amino acid before and after the mutation are shown in Figure 1G and H. Although both leucine and proline are classified as nonpolar aliphatic amino acids, these distinct side chains endow them with different structures and properties. The side chain of leucine is isobutyl, making it a branched amino acid. Proline side chains have a unique circular structure, which gives them special conformational rigidity relative to other amino acids, thereby affecting the secondary structure of proteins.

Besides, a novel variant, c.9814del(p.Arg3272ValfsTer44), was identified in exon 29 of the *PKD1*. This mutation was not only identified in the proband but also her relatives, indicating that this mutation has family inheritance. Two hydrogen bonds are formed between the arginine at position 3272 and the glutamine at position 3100, which become valine after mutation, and the hydrogen bond is broken, resulting in structural changes. In addition, the side chain of arginine is a guanidine group and is a polar amino acid. Valine, on the other hand, contains a nonpolar side chain that is not charged and is hydrophobic, which may affect the folding of PC1 (Fig. 1I, J). The clinical phenotype of the subject is highly consistent with PKD. In addition, through the verification of the subject and her relatives, the subject and her brother who was clinically diagnosed with PKD, and her son clinically suspected of PKD all carried the heterozygous variant, so the variant may exist lineage co-separation in this family.

In conclusion, we used an ion semiconductor sequencing technology, in contrast with traditional, complex, time-consuming, and low-throughput sequencing, to achieve DNA analysis and direct sequencing.^4^ For probands who have been tested and have (potentially) pathogenic variants, specific diagnostic and therapeutic measures can be taken based on their situations. In addition, considering that the proband is of reproductive age, if they have reproductive needs, prenatal diagnosis or preimplantation genetic diagnosis can be offered to guide them and reduce the risk of transmitting the disease to their offspring.^5^ For older probands who carry (potentially) pathogenic variants, it is recommended that their direct relatives undergo screening for these (potentially) pathogenic variants to exclude relatives with low disease risk who do not require clinical follow-up, and to help identify relatives at high risk of disease for early diagnosis, prevention, and treatment.

## Ethics declaration

This study received approval from the Ethics Committee of Shanxi Provincial People's Hospital (2022-275) in accordance with the Declaration of Helsinki. Written informed consent was obtained from all participants, and ethical considerations were diligently observed to safeguard the privacy of each subject.

## Author contributions

Kaili Qin: visualization, writing-original draft, formal analysis; Qian Wang: data curation, investigation, methodology; Jianbo Qing: conceptualization; Yaheng Li: supervision; Hao Gong: resources; Zhijian Zha: visualization; Bingrui Zhou: funding acquisition; Yafeng Li: conceptualization, funding acquisition, project administration. All authors approved the submission of the final version.

## Conflict of interests

The authors declared no conflict of interests.

## Funding

This research was funded by the 10.13039/501100001809National Science Foundation of China (No. 82170716, 81870333, 82100821), the Key Laboratory Construction Plan Project of Shanxi Provincial Health Commission (China) (No. 2020SYS01), the Key Project of Shanxi Provincial Health Commission (China) (No. 2020XM21), and the 10.13039/501100013317Key Research and Development Project of Shanxi Province, China (No. 201903D321086).

## Data availability

Supplementary documents contain additional data generated during this study. For further inquiries regarding detailed data, please feel free to contact the corresponding author.
