# Supplementary material for: Analysis of mutations in Chinese patients with polycystic kidney disease by targeted exome sequencing
Source: Genes Dis. 2024 Feb 28;12(1):101246. doi: 10.1016/j.gendis.2024.101246 (PMC11490921; doi:10.1016/j.gendis.2024.101246)
Supplement: Multimedia component 1 [file mmc1.docx]

Supplementary Materials for

**Analysis of mutations in Chinese Patients with**

**Polycystic Kidney Disease by Targeted Exome Sequencing**

Kaili Qin^1†^, Qian Wang^2†^, Jianbo Qing^1^, Yaheng Li^2^, Hao Gong^3^, Zhijian Zha^7^, Yafeng Li^1,4,5,6, *^

^1^ Department of Nephrology, Shanxi Provincial People’s Hospital (Fifth Hospital) of Shanxi Medical University, Taiyuan 030012, China

^2^ Shanxi Provincial Key Laboratory of Kidney Disease, Shanxi Provincial People's Hospital, Taiyuan 030012, China

^3^ Department of Biochemistry & Molecular Biology, Shanxi Medical University, Taiyuan 030001, Shanxi, China

^4^ Department of Nephrology, Hejin municipal People’s Hospital, Yuncheng 043300, China

^5^ Core Laboratory, Shanxi Provincial People’s Hospital (Fifth Hospital) of Shanxi Medical University, Taiyuan 030012, China

^6^ Academy of Microbial Ecology, Shanxi Medical University, Taiyuan 030012, China

^7^ Third Clinical School, Shanxi University of Traditional Chinese Medicine; No. 89, Sec. 1, Jinzi Road, Wanbailin District, Taiyuan City, Shanxi Province, China

^†^ These authors contributed equally to this work.

^*^ Correspondence: Email: dr.yafengli@gmail.com; Tel: +13935151151

**This file includes:**

Materials and Methods

Table S1 to S7

**Materials and methods**

**Sample collection, extraction and quantification**

For each participant, 3 mL of peripheral blood was collected into a vacuum-extraction blood tubes (EDTA contained). The DNA extraction was followed as the operating instructions offered by Xi 'an Tianlong Technology Co., LTD, with Ex-DNA whole blood genome kit 3.0 and automatic nucleic acid extractor NP968-C. The quantification of extracted DNA was determined by Qubit dsDNA HS Assay Kit (Q32854, Thermo Fisher Scientific) and Qubit 4.0. The qualification of DNA was detected by Nanodrop One (Thermo Fisher Scientific).

**Library Preparation**

*Multiplex polymerase chain reaction (MPCR).* Before carry out MPCR program, dilute each DNA sample to 5 ng/µL. Mix 2 μL of PCR master mix, 5 μL of primer pool 1 or pool 2, 2 μL of DNA sample and 1 μL of nuclease-free ddH_2_O to form two reaction systems for each sample. Then perform a PCR reaction procedure as below: enzyme activated at 99 °C for 2 min, followed by 14 cycles of denature step at 99 °C for 15 sec and anneal/extend step at 60 °C for 8 min. Hold at 10 °C.

*Digestive primer.* Combine the two PCR reaction systems into one PCR tube. Add 2 μL of digest solution into above reaction system and mix well. Digest the primers at 50 °C, 55 °C, 60 °C for 20min each. Hold at 10 °C for less than one hour.

*Connect the sample labels.* Add 4 μL of ligation buffer, 2 μL of index and 2 μL of ligase into above digested product and then mix well. The label procedure was 22 °C for 30 min, followed by 72 °C for 10 min. Hold at 10 °C for less than one hour.

*Clean up the library.* Transfer all the labeled DNA into an Eppendorf LoBind® Tubes and add 45 μL (1.5 ×) of magnetic beads. Mix well and incubate at room temperature for 5 min. After that, place the tubes on a magnetic stand until the solution is clear (about 2 min) and discard the supernatant carefully. Add 150 μL of freshly prepared 70% ethanol to each tube with keep tubes on the magnetic stand. Slowly pipette and carefully remove the supernatant. Wash twice. To dry the beads, open the lid of tubes, keep them on the magnetic stand, remove all liquid, and dry until the surface of the beads is not shiny (about 2 min) at room temperature. Elute the DNA from the beads with 40 μL TE buffer, remove tubes from the magnetic stand and pipette up and down to mix well. Incubate at room temperature for 2 min and then move tubes to the magnetic stand staying 5 min or until the solution change to clear. Transfer the supernatant to a new PCR tube and label the tubes. Libraries can be stored at -20°C.

**Library quantification and sequencing**

The TaqMan qPCR method are performed to quantify of libraries by using Ion Library Quantitation Kit (4468802, Thermo Fisher Scientific).

According to the quantification results, dilute each library to 100 pM. Aliquot same volume of diluted DNA from each library and mix aliquots for pooling libraries. Prepare the templates on Ion OneTouch 2 and Ion OneTouch ES. Then perform the sequencing on Ion Gene Studio S5 System.

**Data analysis and Sanger sequencing**

Bioinformatics tools were used for raw data processing, base detection, sequence alignment, and mutation search. Data annotation by variant analysis tools included conservatism of nucleotides and amino acids, biochemical properties of amino acid substitution, population frequency, and prediction of functional effects. Data analysis focused on the insertions or deletions of small fragment, changes in splicing site, and nonsynonymous changes. The following population and disease related databases used to search reported genetic variants and polymorphisms were as follow: population databases including dbSNP, ExAC, GenomAD, ESP, and the 1000 Genomic Database, disease databases including the OMIM database, HGMD and the ClinVar comprehensive database, and online literature search engines such as PubMed. The variations were then further filtered according to the applicable genetic model. If family member information was available, co-isolation studies could be conducted. Variant interpretation was based on clinical, family and testing information provided by the subject, as well as knowledge of genes and variant sites, including screening and analysis of variant sites related to the subject's clinical phenotype. All relevant results were confirmed by Sanger sequencing.

| No. | Gene | Method | Participants | Region | Novel Mutation | Reference |
| --- | --- | --- | --- | --- | --- | --- |
| 1 | *PKD1* and *PKD2* | Targeted NGS, Sanger sequence | 48 | Spain | - | Trujillano et al. 2014^1^ |
| 2 | *PKD1* and *PKD2* | Targeted next-generation sequence, Sanger sequence | 7 | China | 5 | Yang et al. 2014^2^ |
| 3 | *PKD1* and *PKD2* | Whole exome sequence, MLPA, direct sequence | 20 | South Korea | 10 | Rihwa Choi 2014^3^ |
| 4 | *PKD1* and *PKD2* | LR-PCR, Illumina Sequence (all exonic sequences and flanking intronic regions), Sanger sequence | 25 | America | 3 | Tan et al. 2014^4^ |
| 5 | *PKD1* and *PKD2* | Whole exome sequence, MLPA, direct sequence | 56 | Czech | 25 | Lena Obeidova 2014^5^ |
| 6 | *PKD1*, *PKD2*, *HNF1B* and *PKHD1* | Direct sequence, MLPA, quantitative fluorescent multiplex PCR | 42 | France and Tunisia | - | Audrezet et al. 2016^6^ |
| 7 | *PKD1* and *PKD2* | Sanger sequence, MLPA, RNA | 643 | Italy | 452 | Carrera et al. 2016^7^ |
| 8 | *PKD1*, *PKD2* and *PKHD1* | Ion-Torrent PGM Sequence | 18 | Saudi Arabia | 3 | Edrees et al. 2016^8^ |
| 9 | *PKD1* and *PKD2* | Targeted NGS | 148 | China | 76 | Jin et al. 2016^9^ |
| 10 | *PKD1* and *PKD2* | NGS (Ion Xpress Plus Fragment Library Kit), Sanger sequence, MLPA | 101 | Japan | 60 | Kinoshita et al. 2016^10^ |
| 11 | *PKD1*, *PKD2*, *HNF1B*, *PKHD1*, *SEC63*, *PRKCSH*, *TSC1*, *TSC2* and *OFD1* | Illumina sequence, WGS, Sanger sequence | 28 | Australia | 1 | Mallawaarachchi et al. 2016^11^ |
| 12 | *PKD2* | Automated DNA sequence | 84 | India | 10 | Raj, Singh et al. 2017^12^ |
| 13 | *PKD1* and *PKD2* | Targeted sequence capture and sequence, Sanger sequence | 9 | China | 1 | Sha et al. 2017^13^ |
| 14 | *PKD1*, *PKD2*, *PKHD1*, *HNF1B*, *NPHP1*, *UMOD*, *TSC2*, *COL4A5*, *COL4A3*, *NPHS1*, *NPHS2*, *WT1*, *INF2*, *TRPC6* and *GLA* | Illumina sequence (All exons and exon-intron boundaries) | 135 | Spain | 86 | Bullich et al. 2018^14^ |
| 15 | *PKD1* and *PKD2* | Targeted Next Generation Sequence, Sanger sequence | 9 families | Iran | 3 | Ranjzad et al. 2018^15^ |
| 16 | *PKD1* and *PKD2* | Sanger sequence, MLPA | 18 | Tunisia | 2 | Abdelwahed et al. 2018^16^ |
| 17 | *PKD1* and *PKD2* | LR-PCR, Targeted NGS, Sanger sequence, MLPA | 120 | China | 55 | Xu et al. 2018^17^ |
| 18 | *PKD1* and *PKD2* | Sanger sequence, targeted exome Sequencing, MLPA | 749 | South Korea | 234 | Kim et al. 2019^18^ |
| 19 | *PKD1* and *PKD2* | Whole exome sequence, Illumina sequence, Sanger sequence | 29 | China | 1 | Dong et al. 2019^19^ |
| 20 | PKD1, PKD2 and HNF1B | Sanger sequence, NextSeq 500 tabletop sequencer, WES, MLPA | 47 | China | 18 | Wang et al. 2019^20^ |
| 21 | *PKD1* and *PKD2* | NGS using a hybridization‑based target enrichment method, LR-PCR, Sanger sequence, MLPA | 111 | Japan | 16 | Mochizuki et al. 2019^21^ |
| 22 | *PKD1*, *PKD2*, *GANAB*, *DNAJB11*, *LRP5*, *PMM2*, *PRKCSH*, *SEC63*, *SEC61B*, *ALG8*, *PKHD1*, *DZIP1L*, *HNF1B*, *UMOD*, *INF2* and *REN* | Sanger sequence, MLPA | 212 | Italy | 80 | Mantovani et al. 2020^22^ |
| 23 | *PKD1*, *PKD2* and *GANAB* | Sanger sequence, Targeted NGS, MLPA | 147 | Danish | 41 | Nielsen et al. 2021^23^ |
| 24 | *PKD1*, *PKD2*, *GANAB*, *HNF1B*, *TSC1*, *TSC2*, *OFD1*, *UMOD*, *PKHD1*, *DNAJB11*, *DZIP1L*, *SEC63* and *PRKCSH* | whole-genome and Sanger sequencing, MLPA, LR-PCR | 186 | Australia | - | Mallawaarachchi et al. 2021^24^ |
| 25 | *PKD1*, *PKD2*, *COL4A1*, *DNAJB11*, *GANAB*, *HNF1B*, *REN* and *UMOD* | targeted sequencing | 56 | Canada | - | Elliott et al. 2021^25^ |
| 26 | *PKD1*, *PKD2*, *PKHD1*, *TSC1*, *TSC2*, *VHL*, *HNF1B*, *NPHP1*, *NPHP3* and *NPHP4* | NGS, LR-PCR, direct sequence, MLPA | 26 | South Korea | 8 | Kim et al. 2021^26^ |
| 27 | *PKD1* and *PKD2* | NGS (target-gene), Sanger sequence, MLPA | 21(6 families) | China | 3 | Hanlu Wang etal. 2022^27^ |
| 28 | *PKD1*, *PKD2* and *GANAB* | Sanger sequence, MLPA | 19 | Tunisia | 3 | Abdelwahed et al. 2022^28^ |
| 29 | *PKD1*, *PKD2*, *PKHD1*, *PKD1L3*, *EGF*, *CFTR* and *TSC2* | massive parallel sequencing NGS (Ion PI Hi-Q Sequencing 200 Kit and the Ion PI Chip Kit v3) | 11 | Kingdom of Saudi Arabia | 3 | Alzahrani et al. 2022^29^ |
| 30 | *AHI1*, *ALG8*, *ANKS6*, *BBIP1*, *BBS1*, *BS2*, *BBS10*, *BBS12*, *C5orf42*, *DNAJB11*, *DZIP1L*, *LRP5*, *TSC2*, *TTC21B*, *TTC8*, *UMOD*, *WDPCP*, etc. | exome sequencing, Sanger sequence, MLPA | 50 | Japan | 34 | Suzuki et al. 2023^30^ |

**Table S1.** Information on gene sequencing of polycystic kidney in the past decade.

**Table S2.** Information about the 15 genes involved in sequencing.

| Gene | Type | Location | Protein | Functions | Reported sites number |
| --- | --- | --- | --- | --- | --- |
| *PKD1* | ADPKD | 16p13.3 | Polycystin-1(PC1) | It acts as a calcium-permeable cation channel. Involved in tubular development. | 2054(2023.1) |
| *PKD2* | ADPKD | 4q22.1 | Polycystin-2(PC2) | Plays the role of calcium-permeable cation channel. Interacts with polycystin 1 and is involved in tubular development. | 347(2023.1) |
| *GANAB* | ADPKD | 11q12.3 | Catalytic subunit of glucosidase II | Participate in the processing, folding and maturation of PC1 and PC2. | 20(2023.1) |
| *DNAJB11* | ADPKD | 3q27.3 | DNAJ heat shock protein family (Hsp40) member B11 | As a co-companion of HSPA5, involved in protein folding, transport and degradation. | 6(2023.1) |
| *ALG8* | ADPKD | 11q14.1 | Alpha-1,3-glucosyltransferase | A second glucose residue is added to the lipid-linked oligosaccharide precursor to achieve N-linked glycosylation of proteins. | 22(2023.1) |
| *PKHD1* | ARPKD | 6p12.3-p12.2 | Fibrocystin protein | Promotes ciliogenesis in renal epithelial cells. Involves cell-to-cell adhesion, rejection, and proliferation. | 572(2023.1) |
| *DZIP1L* | ARPKD | 3q22.3 | Cilium assembly protein DZIP1L | Involved in primary cilium formation. Probably acts as a transition zone protein required for localization of PC1 and PC2 to the ciliary membrane. | 5(2023.1) |
| *ANKS6* | Nephronophthisis | 9q22.33 | ankyrin repeat and sterile alpha motif domain containing 6 | Plays a role in kidney and cardiovascular development. | 14(2023.1) |
| *COL4A3* | Alport syndrome | 2q36.3 | collagen type IV alpha 3 chain | As the main structural component of the basement membrane. | 284(2023.1) |
| *COL4A5* | Alport syndrome | Xq22.3 | collagen type IV alpha 5 chain | As the main structural component of the basement membrane. | 1140(2023.1) |
| *HNF1B* | renal cysts and diabetes syndrome | 17q12 | hepatocyte nuclear factor 1-beta | Transcription factor, binds to the FPC element, transcriptional activity is increased by coactivator PCBD1. | 260(2023.1) |
| *LRP5* | Polycystic liver disease type IV | 11q13.2 | LDL receptor related protein 5 | As a co-receptor with Frizzled protein family members for transducing signals by Wnt proteins. | 267(2023.1) |
| *TSC1* | tuberous sclerosis and lymphangioleiomyomatosis | 9q34.13 | TSC complex subunit 1/hamartin | As a co-chaperone for HSP90 facilitating HSP90, negative regulator of mTORC1, as a tumor suppressor. | 418(2023.1) |
| *TSC2* | tuberous sclerosis and lymphangioleiomyomatosis | 16p13.3 | TSC complex subunit 2/ tuberin | As a GTPase-activating protein (GAP) for the small GTPase RHEB, a direct activator of the protein kinase activity of mTORC1. | 1309(2023.1) |
| *VHL* | von Hippel-Lindau syndrome, pheochromocytoma, erythrocytosis, renal cell carcinoma, and cerebellar hemangioblastoma | 3p25.3 | von Hippel-Lindau tumor suppressor (a component of a ubiquitination complex) | Involved in the ubiquitination and subsequent proteasomal degradation, as a target recruitment subunit, involved in transcriptional repression. | 624(2023.1) |

**Table S3.** Statistics regarding the diagnoses of PKD among participants.

|  | Diagnosed | Suspected^a^ | Undiagnosed | Total |
| --- | --- | --- | --- | --- |
| Proband | 13 | 0 | 0 | 13 |
| Relative | 12 | 10 | 2 | 24 |
| Singleton | 58 | 0 | 0 | 58 |
| Total | 83 | 10 | 2 | 95 |

^a^ The participants did not have a confirmed diagnosis of PKD through ultrasound, but had a positive family history of PKD.

**Table S4. Clinical information pertaining to the participants.**

| ID | Creatinine  (μmol/L) | eGFR (mL/min/1.73m^2^) | CKD stage | ID | Creatinine  (μmol/L) | eGFR (mL/min/1.73m^2^) | CKD stage |
| --- | --- | --- | --- | --- | --- | --- | --- |
| 1 | 349.5 | 11.32 | G5 | 49 | 614.63 | 6.03 | G5 |
| 2 | 529.7 | 8.36 | G5 | 50 | 691.19 | 7.13 | G5 |
| 3 | 467.4 | 11.08 | G5 | 51 | 219.25 | 27.1 | G4 |
| 4 | 452.7 | 8.35 | G5 | 52 | 825.63 | 5.89 | G5 |
| 5 | 510.3 | 11.45 | G5 | 53 | 388.48 | 10.35 | G5 |
| 6 | 408.98 | 9.94 | G5 | 54 | 233.05 | 18.3 | G4 |
| 7 | - | - | G4 | 55 | 100.7 | 67.25 | G2 |
| 8 | 120.8 | 39.72 | G3b | 56 | 469.22 | 8.13 | G5 |
| 9 | - | - | G5 | 57 | 464.26 | 11.03 | G5 |
| 10 | - | - | G5 | 58 | 493.42 | 11.32 | G5 |
| 11 | 572.1 | 8.83 | G5 | 59 | 255.83 | 20.84 | G4 |
| 12 | - | - | G4 | 60 | 530.7 | 9.18 | G5 |
| 13 | 92.5 | 56.65 | G3a | 61 | 291.53 | 20.04 | G4 |
| 14 | 378.42 | 14.54 | G5 | 62 | 969.75 | 4.89 | G5 |
| 15 | - | - | G5 | 63 | 463.35 | 11.02 | G5 |
| 16 | 671.3 | 5.63 | G5 | 64 | 419.92 | 9.37 | G5 |
| 17 | 602.51 | 7.11 | G5 | 65 | 435.66 | 12.18 | G5 |
| 18 | 451.6 | 8.83 | G5 | 66 | 89.31 | 75.07 | G2 |
| 19 | - | - | G5 | 67 | 512.46 | 7.79 | G5 |
| 20 | 619.87 | 6.93 | G5 | 68 | 71.13 | 74.53 | G2 |
| 21 | - | - | G3b | 69 | 91.92 | 57.06 | G3a |
| 22 | 312.6 | 15.16 | G4 | 70 | 356.78 | 11.5 | G5 |
| 23 | 506.34 | 10.31 | G5 | 71 | 467.81 | 7.82 | G5 |
| 24 | - | - | G5 | 72 | 526.04 | 9.61 | G5 |
| 25 | 204.8 | 31.89 | G3b | 73 | 287.87 | 19.08 | G4 |
| 26 | 529.3 | 9.76 | G5 | 74 | 308.24 | 13.05 | G5 |
| 27 | 359.6 | 11.67 | G5 | 75 | 102 | 50.17 | G3a |
| 28 | - | - | G4 | 76 | 603.1 | 6 | G5 |
| 29 | 549.5 | 6.99 | G5 | 77 | 53.59 | 109.41 | G1 |
| 30 | 207.68 | 28.44 | G4 | 78 | 899.27 | 4.02 | G5 |
| 31 | - | - | G5 | 79 | 88.74 | 91.4 | G1 |
| 32 | 378.5 | 15.87 | G4 | 80 | 650.18 | 7.45 | G5 |
| 33 | 621.8 | 9.18 | G5 | 81 | 534.67 | 10.37 | G5 |
| 34 | 489.5 | 9.93 | G5 | 82 | 427.59 | 8.97 | G5 |
| 35 | 209 | 28.24 | G4 | 83 | 723.32 | 5.09 | G5 |
| 36 | 386.98 | 10.86 | G5 | 84 | 863.07 | 5.7 | G5 |
| 37 | 214.67 | 22.41 | G4 | 85 | 90.5 | 76.08 | G2 |
| 38 | 451.98 | 9.77 | G5 | 86 | 367.95 | 13.98 | G5 |
| 39 | 564.61 | 6.72 | G5 | 87 | 466.63 | 11.18 | G5 |
| 40 | 104.56 | 61.59 | G2 | 88 | 398.27 | 10.13 | G5 |
| 41 | 532.67 | 9.73 | G5 | 89 | 428.4 | 12.75 | G5 |
| 42 | 398.45 | 9.7 | G5 | 90 | 163.52 | 38.01 | G3b |
| 43 | 549.67 | 9.35 | G5 | 91 | 304.7 | 15.83 | G4 |
| 44 | 587.17 | 6.77 | G5 | 92 | 489.54 | 10.69 | G5 |
| 45 | 562.76 | 6.47 | G5 | 93 | 100.87 | 49.43 | G3a |
| 46 | 208.67 | 22.79 | G4 | 94 | 202.76 | 24.21 | G4 |
| 47 | 432.17 | 12 | G5 | 95 | 369.38 | 11.41 | G5 |
| 48 | 421.39 | 13.65 | G5 |  |  |  |  |

**Table S5.** Results of gene sequencing for 95 participants

| **ID** | **Sex** | **Age** | **Relationship** | **PKD** | **Gene** | **Transcript accession no.** | **Position** | **cDNA variant**  **(Protein variant)** | **Pathogenicity** | **Described**  **/Novel** | | |
| --- | --- | --- | --- | --- | --- | --- | --- | --- | --- | --- | --- | --- |
| **Singleton** | | | | | | | | | | | | |
| 1 | F | 67 | - | Yes | - |  |  |  |  |  | | |
| 2 | F | 28 | - | Yes | *PKHD1* | NM_138694 | chr6:51921776 | c.1514T>G(p.Val505Gly) | VUS2 | Novel | | |
| 2 | F | 28 | - | Yes | *PKD2* | NM_000297 | chr4:88973158 | c.1564A>G(p.Ile522Val) | VUS3 | Described | | |
| 3 | M | 62 | - | Yes | *PKD2* | NM_000297 | chr4:88959394 | c.844-9T>A(-) | VUS2 | Novel | | |
| 3 | M | 62 | - | Yes | *PKD1* | NM_001009944 | chr16:2159344 | c.5824C>T(p.Arg1942Cys) | VUS3 | Described | | |
| 4 | F | 69 | - | Yes | *COL4A3* | NM_000091 | chr2:228163439 | c.3794del(p.Gly1265AlafsTer2) | LP | Novel | | |
| 25 | M | 37 | - | Yes | *PKD2* | NM_000297 | chr4:88977237 | c.1717-1G>A(-) | LP | Described | | |
| 25^a^ | M | 37 | - | Yes | *LRP5* | NM_002335 | chr11:68115513 | c.290C>T(p.Ala97Val) | LB | Described | | |
| 26 | M | 57 | - | Yes | *COL4A5* | NM_033380 | chrX:107935999 | c.4550G>A(p.Arg1517His) | VUS3 | Described | | |
| 26 | M | 57 | - | Yes | *DZIP1L* | NM_173543 | chr3:137790602 | c.1498C>T(p.Arg500Trp) | VUS3 | Described | | |
| 32 | M | 35 | - | Yes | *PKHD1* | NM_138694 | chr6:51910887 | c.2507T>C(p.Val836Ala) | P | Described | | |
| 32 | M | 35 | - | Yes | *PKHD1* | NM_138694 | chr6:51611616 | c.9901G>T(p.Glu3301Ter) | P | Described | | |
| 32 | M | 35 | - | Yes | *PKHD1* | NM_138694 | chr6:51483985 | c.12119A>C(p.Gln4040Pro) | VUS3 | Novel | | |
| 35 | M | 60 | - | Yes | - |  |  |  |  |  | | |
| 39 | F | 57 | - | Yes | - |  |  |  |  |  | | |
| 40 | M | 66 | - | Yes | *PKD1* | NM_001009944 | chr16:2140491 | c.12239G>A(p.Trp4080Ter) | P | Described | | |
| 41 | M | 56 | - | Yes | *PKD1* | NM_001009944 | chr16:2158497 | c.6657_6671del (p.Arg2220_Pro2224del) | LP | Described | | |
| 42 | F | 68 | - | Yes | *PKD1* | NM_001009944 | chr16:2139817 | c.12823G>A(p.Ala4275Thr) | VUS2 | Described | | |
| 42 | F | 68 | - | Yes | *PKHD1* | NM_138694 | chr6:51512879 | c.11348C>T(p.Pro3783Leu) | VUS2 | Novel | | |
| 43 | M | 57 | - | Yes | *PKD1* | NM_001009944 | chr16:2142075 | c.11384G>C(p.Trp3795Ser) | VUS2 | Novel | | |
| 48^a^ | F | 40 | - | Yes | *DZIP1L* | NM_173543 | chr3:137783495 | c.2117C>A(p.Ala706Asp) | VUS3 | Described | | |
| 48^a^ | F | 40 | - | Yes | *DZIP1L* | NM_173543 | chr3:137796432 | c.1331C>G(p.Ala444Gly) | VUS2 | Novel | | |
| 49 | F | 60 | - | Yes | *PKD1* | NM_001009944 | chr16:2160153 | c.5014_5015del (p.Arg1672GlyfsTer98) | P | Described | | |
| 49 | F | 60 | - | Yes | *LRP5* | NM_002335 | chr11:68216333 | c.4643G>T(p.Cys1548Phe) | VUS3 | Described | | |
| 50 | M | 59 | - | Yes | *PKD1* | NM_001009944 | chr16:2140185 | c.12455A>C(p.Lys4152Thr) | VUS3 | Described | | |
| 50 | M | 59 | - | Yes | *COL4A5* | NM_033380 | chrX:107834459 | c.1337C>T(p.Pro446Leu) | VUS3 | Novel | | |
| 51 | M | 56 | - | Yes | *PKD2* | NM_000297 | chr4:88964463 | c.1173T>G(p.Tyr391Ter) | LP | Novel | | |
| 51 | M | 56 | - | Yes | *ANKS6* | NM_173551 | chr9:101536290 | c.1690C>A(p.Pro564Thr) | VUS3 | Described | | |
| 51 | M | 56 | - | Yes | *TSC1* | NM_000368 | chr9:135781505 | c.1460C>G(p.Ser487Cys) | VUS3 | Described | | |
| 52^a^ | M | 55 | - | Yes | *PKD1* | NM_001009944 | chr16:2152482 | c.9086_9103del  (p.Gly3029_Glu3034del) | VUS1 | Novel | | |
| 52^a^ | M | 55 | - | Yes | *COL4A3* | NM_000091 | chr2:228173943 | c.4664C>T(p.Ala1555Val) | VUS2 | Described | | |
| 53 | F | 57 | - | Yes | - |  |  |  |  |  | | |
| 54 | F | 63 | - | Yes | *PKD1* | NM_001009944 | chr16:2159440 | c.5728del  (p.Leu1910CysfsTer39) | LP | Novel | | |
| 55 | M | 53 | - | Yes | *PKD2* | NM_000297 | chr4:88967920 | c.1449dup  (p.Ile484TyrfsTer42) | LP | Novel | | |
| 55^a^ | M | 53 | - | Yes | *PKD1* | NM_001009944 | chr16:2160498 | c.4670G>T(p.Arg1557Leu) | VUS3 | Described | | |
| 55^a^ | M | 53 | - | Yes | *COL4A3* | NM_000091 | chr2:228159737 | c.3476G>A(p.Arg1159His) | LB | Described | | |
| 56 | F | 64 | - | Yes | - |  |  |  |  |  | | |
| 57 | M | 66 | - | Yes | *PKD1* | NM_001009944 | chr16:2149887 | c.9898G>A(p.Gly3300Arg) | VUS3 | Described | | |
| 58 | M | 41 | - | Yes | - |  |  |  |  |  | | |
| 59 | M | 85 | - | Yes | - |  |  |  |  |  | | |
| 60 | M | 76 | - | Yes | *PKD2* | NM_000297 | chr4:88959523 | c.964C>T(p.Arg322Trp) | P | Described | | |
| 60 | M | 76 | - | Yes | *PKD1* | NM_001009944 | chr16:2159529 | c.5639A>G(p.Asn1880Ser) | VUS3 | Described | | |
| 61 | M | 49 | - | Yes | *PKD1* | NM_001009944 | chr16:2143956 | c.10667_10677delinsAGGGA  (p.Ser3556_His3559delinsTer) | LP | Novel | | |
| 62^a^ | M | 55 | - | Yes | *PKD1* | NM_001009944 | chr16:2159199 | c.5968_5969del(p.Arg1990GlufsTer59) | P | Described | | |
| 62 | M | 55 | - | Yes | *PKD1* | NM_001009944 | chr16:2140032 | c.12608G>A(p.Arg4203Gln) | VUS3 | Described | | |
| 62^a^ | M | 55 | - | Yes | *PKHD1* | NM_138694 | chr6:51893003 | c.3511C>G(p.Leu1171Val) | VUS3 | Described | | |
| 63 | M | 67 | - | Yes | *PKD1* | NM_001009944 | chr16:2142574 | c.11176T>C(p.Trp3726Arg) | LP | Described | | |
| 64 | F | 60 | - | Yes | *PKD1* | NM_001009944 | chr16:2160153 | c.5014_5015del  (p.Arg1672GlyfsTer98) | P | Described | | |
| 65 | M | 58 | - | Yes | - |  |  |  |  |  | | |
| 66 | M | 61 | - | Yes | - |  |  |  |  |  | | |
| 67^a^ | F | 48 | - | Yes | *PKD1* | NM_001009944 | chr16:2160153 | c.5014_5015del  (p.Arg1672GlyfsTer98) | P | Described | | |
| 68 | F | 53 | - | Yes | - |  |  |  |  |  | | |
| 69 | F | 46 | - | Yes | *PKD1* | NM_001009944 | chr16:2159137 | c.6031C>T(p.Gln2011Ter) | P | Described | | |
| 70 | F | 55 | - | Yes | *PKD1* | NM_001009944 | chr16:2156212 | c.7581_7582dup  (p.Tyr2528SerfsTer93) | LP | Novel | | |
| 70 | F | 55 | - | Yes | *PKD1* | NM_001009944 | chr16:2140185 | c.12455A>C(p.Lys4152Thr) | VUS3 | Described | | |
| 71 | F | 79 | - | Yes | *PKD2* | NM_000297 | chr4:88973310 | c.1716G>C(p.Lys572Asn) | VUS2 | Novel | | |
| 72 | M | 64 | - | Yes | *PKD2* | NM_000297 | chr4:88989214 | c.2522+1G>C(-) | VUS1 | Novel | | |
| 73 | M | 67 | - | Yes | - |  |  |  |  |  | | |
| 74 | F | 68 | - | Yes | *PKD1* | NM_001009944 | chr16:2156116 | c.7650_7679del  (p.Leu2551_Gly2560del) | VUS2 | Novel | | |
| 75 | F | 48 | - | Yes | *PKD1* | NM_001009944 | chr16:2150477 | c.9488A>T(p.Asn3163Ile) | VUS2 | Novel | | |
| 76^a^ | F | 69 | - | Yes | *DZIP1L* | NM_173543 | chr3:137786533 | c.1841dup  (p.Phe615ValfsTer4) | LP(AR) | Novel | | |
| 77 | F | 40 | - | Yes | *PKD1* | NM_001009944 | chr16:2161791 | c.3376dup  (p.Leu1126ProfsTer10) | P/LP? | Novel | | |
| 77 | F | 40 | - | Yes | *PKHD1* | NM_138694 | chr6:51889377 | c.5231A>G(p.Asn1744Ser) | VUS3 | Described | | |
| 77 | F | 40 | - | Yes | *DZIP1L* | NM_173543 | chr3:137796336 | c.1422+5G>A(-) | VUS3 | Described | | |
| 78 | F | 51 | - | Yes | *ALG8* | NM_024079 | chr11:77832213 | c.376A>G(p.Lys126Glu) | VUS3 | Described | | |
| 78 | F | 51 | - | Yes | *TSC2* | NM_000548 | chr16:2134692 | c.4469A>G(p.Glu1490Gly) | VUS3 | Described | | |
| 78 | F | 51 | - | Yes | *DZIP1L* | NM_173543 | chr3:137796336 | c.1422+5G>A(-) | VUS3 | Described | | |
| 79 | M | 24 | - | Yes | *PKD2* | NM_000297 | chr4:88989091 | c.2401_2402del  (p.Ser801GlnfsTer10) | LP | Novel | | |
| 80 | M | 67 | - | Yes | *PKHD1* | NM_138694 | chr6:51701234 | c.8141G>A(p.Arg2714Gln) | VUS3 | Described | | |
| 80 | M | 67 | - | Yes | *TSC1* | NM_000368 | chr9:135781265 | c.1700C>T(p.Ala567Val) | VUS3 | Described | | |
| 81 | M | 40 | - | Yes | *PKD1* | NM_001009944 | chr16:2152250 | c.9206_9209del  (p.Pro3069GlnfsTer4) | LP | Novel | | |
| 82^a^ | F | 67 | - | Yes | *LRP5* | NM_002335 | chr11:68174186 | c.1996G>A(p.Asp666Asn) | VUS3 | Described | | |
| 83 | F | 55 | - | Yes | *PKD1* | NM_001009944 | chr16:2159142 | c.6026C>A(p.Ser2009Ter) | P | Novel | | |
| 84 | M | 50 | - | Yes | *PKD1* | NM_001009944 | chr16:2140563 | c.12166_12167del  (p.Trp4056GlufsTer100) | LP | Described | | |
| 84 | M | 50 | - | Yes | *PKHD1* | NM_138694 | chr6:51656139 | c.8335T>G(p.Phe2779Val) | VUS3 | Described | | |
| 85 | M | 53 | - | Yes | *PKD1* | NM_001009944 | chr16:2162883 | c.3067C>T(p.Gln1023Ter) | P | Described | | |
| 86 | M | 77 | - | Yes | *PKD1* | NM_001009944 | chr16:2142080 | c.11379del(p.Thr3794ArgfsTer32) | P | Described | | |
| 87 | M | 60 | - | Yes | *PKD1* | NM_001009944 | chr16:2147228 | c.10420C>T(p.Gln3474Ter) | P | Described | | |
| 88 | F | 55 | - | Yes | *PKD1* | NM_001009944 | chr16:2165489 | c.1987del(p.Gln663ArgfsTer122) | LP | Described | | |
| 89 | M | 51 | - | Yes | *PKD1* | NM_001009944 | chr16:2159316 | c.5852G>A(p.Arg1951Gln) | VUS2 | Described | | |
| 90 | M | 56 | - | Yes | *PKD2* | NM_000297 | chr4:88940696 | c.682C>G(p.Leu228Val) | VUS3 | Novel | | |
| 90 | M | 56 | - | Yes | *PKD2* | NM_000297 | chr4:88989214 | c.2522+1G>A(-) | VUS2 | Novel | | |
| 90 | M | 56 | - | Yes | *PKHD1* | NM_138694 | chr6:51890823 | c.3785G>A(p.Ala1262Val) | B | Described | | |
| 90 | M | 56 | - | Yes | *COL4A5* | NM_033380 | chrX:107865062 | c.2707C>T(p.Pro903Ser) | VUS3 | Novel | | |
| 94 | F | 35 | - | Yes | *HNF1B* | NM_000458 | chr17:36070671 | c.1046del(p.Gly349GlufsTer27) | P | Described | | |
| 95 | F | 47 | - | Yes | *PKD1* | NM_001009944 | chr16:2143104 | c.11017-10C>A(-) | P | Described | | |
| **Family 1** | | | | | | | | | | | | |
| 5 | M | 32 | **Proband** | Yes | *PKHD1* | NM_138694 | chr6:51613004 | c.9410G>A(p.Gly3137Glu) | VUS2 | | Described | |
| 6 | F | 52 | Mother | Yes | *PKHD1* | NM_138694 | chr6:51613004 | c.9410G>A(p.Gly3137Glu) | VUS2 | | Described | |
| 7 | M | 5 | Son | Suspect | *PKHD1* | NM_138694 | chr6:51613004 | c.9410G>A(p.Gly3137Glu) | VUS2 | | Described | |
| **Family 2** | | | | | | | | | | | | |
| 8 | F | 58 | **Proband** | Yes | *COL4A5* | NM_033380 | chrX:107807142 | c.262C>T(p.Pro88Ser) | VUS2 | | Described | |
| 9 | F | 25 | Daughter | Suspect | *COL4A5* | NM_033380 | chrX:107807142 | c.262C>T(p.Pro88Ser) | VUS2 | | Described | |
| 10 | F | 32 | Daughter | Suspect | - |  |  |  |  | | Described | |
| **Family 3** | | | | | | | | | | | | |
| 11 | M | 60 | **Proband** | Yes | - |  |  |  |  | |  | |
| 12 | F | 35 | Daughter | Suspect | - |  |  |  |  | |  | |
| **Family 4** | | | | | | | | | | | | |
| 13 | F | 46 | **Proband** | Yes | *PKD1* | NM_001009944 | chr16:2149971 | c.9814del(p.Arg3272ValfsTer44) | P | | Novel | |
| 14 | M | 54 | Brother | Yes | *PKD1* | NM_001009944 | chr16:2149971 | c.9814del(p.Arg3272ValfsTer44) | P | | Novel | |
| 15 | M | 7 | Son | Suspect | *PKD1* | NM_001009944 | chr16:2149971 | c.9814del(p.Arg3272ValfsTer44) | P | | Novel | |
| **Family 5** | | | | | | | | | | | | |
| 16 | F | 51 | **Proband** | Yes | - |  |  |  |  | |  | |
| 17 | F | 30 | Daughter | Yes | - |  |  |  |  | |  | |
| **Family 6** | | | | | | | | | | | | |
| 18 | F | 53 | **Proband** | Yes | - |  |  |  |  | |  | |
| 19 | F | 32 | Daughter | Suspect | - |  |  |  |  | |  | |
| 20 | F | 29 | Daughter | Yes | - |  |  |  |  | |  | |
| 21 | M | 21 | Son | Suspect | - |  |  |  |  | |  | |
| **Family 7** | | | | | | | | | | | | |
| 22 | F | 30 | **Proband** | Yes | *PKD2* | NM_000297 | chr4:88973194 | c.1600G>C(p.Val534Leu) | VUS2 | | Described | |
| 23 | M | 56 | Father | Yes | *PKD2* | NM_000297 | chr4:88973194 | c.1600G>C(p.Val534Leu) | VUS2 | | Described | |
| 24 | F | 56 | Mother | Suspect | - |  |  |  |  | | Described | |
| **Family 8** | | | | | | | | | | | | |
| 27 | F | 49 | **Proband** | Yes | *PKD1* | NM_001009944 | chr16:2158285 | c.6882_6883del(p.Ser229PhefsTer124) | P | | Described | |
| 27 | F | 49 | **Proband** | Yes | *TSC2* | NM_000548 | chr16:2121610 | c.1939G>A(p.Asp647Asn) | VUS3 | | Described | |
| 27^a^ | F | 49 | **Proband** | Yes | *ANKS6* | NM_173551 | chr9:101536290 | c.1690C>A(p.Pro564Thr) | VUS3 | | Described | |
| 28 | M | 27 | Son | Suspect | *TSC2* | NM_000548 | chr16:2121610 | c.1939G>A(p.Asp647Asn) | VUS3 | | Described | |
| **Family 9** | | | | | | | | | | | | |
| 29 | F | 55 | **Proband** | Yes | *PKD2* | NM_000297 | chr4:88977237 | c.1717-1G>A(-) | LP | | Described | |
| 29 | F | 55 | **Proband** | Yes | *LRP5* | NM_002335 | chr11:68206126 | c.4324G>A(p.Gly1442Ser) | VUS3 | | Described | |
| 30 | M | 60 | Brother | Yes | *PKD2* | NM_000297 | chr4:88977237 | c.1717-1G>A(-) | LP | | Described | |
| 31 | F | 33 | Nephew | Suspect | - |  |  |  |  | |  | |
| **Family 10** | | | | | | | | | | | | |
| 33 | M | 31 | **Proband** | Yes | *PKD1* | NM_001009944 | chr16:2160153 | c.5014_5015del(p.Arg1672GlyfsTer98) | P | | Described | |
| 34 | M | 82 | Father | Yes | - |  |  |  |  | |  | |
| **Family 11** | | | | | | | | | | | | |
| 36^a^ | F | 46 | **Proband** | Yes | *LRP5* | NM_002335 | chr11:68115513 | c.290C>T(p.Ala97Val) | LB | | Described | |
| 37 | F | 37 | Nephew | Yes | - |  |  |  |  | |  | |
| 38 | F | 32 | Nephew | Yes | - |  |  |  |  | |  | |
| **Family 12** | | | | | | | | | | | | |
| 44 | F | 44 | **Proband** | Yes | *PKD1* | NM_001009944 | chr16:2158290 | c.6878C>T(p.Pro2293Leu) | VUS3 | | Described | |
| 44 | F | 44 | **Proband** | Yes | *TSC2* | NM_000548 | chr16:2121610 | c.1939G>A(p.Asp647Asn) | VUS3 | | Described | |
| 45 | F | 70 | Mother | Yes | *PKD1* | NM_001009944 | chr16:2153747 | c.8311G>A(p.Glu2771Lys) | P | | Described | |
| 45 | F | 70 | Mother | Yes | *PKD1* | NM_001009944 | chr16:2158290 | c.6878C>T(p.Pro2293Leu) | VUS3 | | Described | |
| 45 | F | 70 | Mother | Yes | *TSC2* | NM_000548 | chr16:2121610 | c.1939G>A(p.Asp647Asn) | VUS3 | | Described | |
| 46 | F | 40 | Sister | Yes | *PKD1* | NM_001009944 | chr16:2153747 | c.8311G>A(p.Glu2771Lys) | P | | Described | |
| 47 | F | 15 | Daughter | Unknown | *PKD1* | NM_001009944 | chr16:2143832 | c.10801C>(p.Leu3601Phe) | VUS2 | | Described | |
| 47 | F | 15 | Daughter | Unknown | *ANKS6* | NM_173551 | chr9:101544784 | c.1184C>T(p.Thr395Met) | VUS3 | | Described | |
| **Family 13** | | | | | | | | | | | | |
| 91 | F | 28 | Proband | Yes | - |  |  |  |  | | |  |
| 92 | M | 57 | Father | No | - |  |  |  |  | | |  |
| 93 | F | 55 | Mother | No | - |  |  |  |  | | |  |

F, female; M, male; P, pathogenic; LP, likely Pathogenic; VUS, variant of uncertain significance; LB, likely benign; B, benign; ^a^ Sanger sequencing was not performed due to insufficient sample size

**Table S6.** Summary of types of variants in genes.

| Gene | Missense | Nonsense | Frameshift Indel | In-frame Indel | Splicing Site | Total |
| --- | --- | --- | --- | --- | --- | --- |
| *PKD1* | 14 | 6 | 11 | 3 | 1 | 35 |
| *PKD2* | 5 | 1 | 2 | 0 | 4 | 12 |
| *PKHD1* | 10 | 1 | 0 | 0 | 0 | 11 |
| *TSC1* | 2 | 0 | 0 | 0 | 0 | 2 |
| *TSC2* | 2 | 0 | 0 | 0 | 0 | 2 |
| *ALG8* | 1 | 0 | 0 | 0 | 0 | 1 |
| *ANKS6* | 2 | 0 | 0 | 0 | 0 | 2 |
| *COL4A3* | 2 | 0 | 1 | 0 | 0 | 3 |
| *COL4A5* | 4 | 0 | 0 | 0 | 0 | 4 |
| *DZIP1L* | 3 | 0 | 1 | 0 | 1 | 5 |
| *HNF1B* | 0 | 0 | 1 | 0 | 0 | 1 |
| *LRP5* | 4 | 0 | 0 | 0 | 0 | 4 |
| Total | 49 | 8 | 16 | 3 | 6 | 82 |

**Table S7.** Pathogenic classification of participant gene sequencing.

| Gene | P | LP | VUS1 | VUS2 | VUS3 | LB | B | Total |
| --- | --- | --- | --- | --- | --- | --- | --- | --- |
| *PKD1* | 13 | 8 | 1 | 6 | 7 | 0 | 0 | 35 |
| *PKD2* | 1 | 4 | 1 | 4 | 2 | 0 | 0 | 12 |
| *PKHD1* | 2 | 0 | 0 | 3 | 5 | 0 | 1 | 11 |
| *TSC1* | 0 | 0 | 0 | 0 | 2 | 0 | 0 | 2 |
| *TSC2* | 0 | 0 | 0 | 0 | 2 | 0 | 0 | 2 |
| *ALG8* | 0 | 0 | 0 | 0 | 1 | 0 | 0 | 1 |
| *ANKS6* | 0 | 0 | 0 | 0 | 2 | 0 | 0 | 2 |
| *COL4A3* | 0 | 1 | 0 | 1 | 0 | 1 | 0 | 3 |
| *COL4A5* | 0 | 0 | 0 | 1 | 3 | 0 | 0 | 4 |
| *DZIP1L* | 0 | 1 | 0 | 1 | 3 | 0 | 0 | 5 |
| *HNF1B* | 1 | 0 | 0 | 0 | 0 | 0 | 0 | 1 |
| *LRP5* | 0 | 0 | 0 | 0 | 3 | 1 | 0 | 4 |
| Total | 17 | 14 | 2 | 16 | 30 | 2 | 1 | 82 |

P, pathogenic; LP, likely Pathogenic; VUS, variant of uncertain significance; LB, likely benign; B, benign

Rreferences

1. Trujillano D, Bullich G, Ossowski S, et al. Diagnosis of autosomal dominant polycystic kidney disease using efficient PKD1 and PKD2 targeted next-generation sequencing. *Mol Genet Genomic Med.* 2014;2(5):412-421.

2. Yang T, Meng Y, Wei X, et al. Identification of novel mutations of PKD1 gene in Chinese patients with autosomal dominant polycystic kidney disease by targeted next-generation sequencing. *Clin Chim Acta.* 2014;433:12-19.

3. Rihwa Choi HCP, Kyunghoon Lee, Myoung-Gun Lee, Jong-Won Kim, Chang-Seok Ki, Young-Hwan Hwang and Curie Ahn. Identification of novel PKD1 and PKD2 mutations in Korean patients with autosomal dominant polycystic kidney disease. *BMC Med Genet.* 2014.

4. Tan AY, Michaeel A, Liu G, et al. Molecular diagnosis of autosomal dominant polycystic kidney disease using next-generation sequencing. *J Mol Diagn.* 2014;16(2):216-228.

5. Lena Obeidova VE, Jitka Stekrova, Jana Reiterova, Miroslav Merta, Vladimir Tesar, Frantisek Losan and Milada Kohoutova. Novel mutations of PKD genes in the Czech population with autosomal dominant polycystic kidney disease. *BMC Med Genet.* 2014.

6. Audrezet MP, Corbiere C, Lebbah S, et al. Comprehensive PKD1 and PKD2 Mutation Analysis in Prenatal Autosomal Dominant Polycystic Kidney Disease. *J Am Soc Nephrol.* 2016;27(3):722-729.

7. Carrera P, Calzavara S, Magistroni R, et al. Deciphering Variability of PKD1 and PKD2 in an Italian Cohort of 643 Patients with Autosomal Dominant Polycystic Kidney Disease (ADPKD). *Sci Rep.* 2016;6:30850.

8. Edrees BM, Athar M, Al-Allaf FA, et al. Next-generation sequencing for molecular diagnosis of autosomal recessive polycystic kidney disease. *Gene.* 2016;591(1):214-226.

9. Jin M, Xie Y, Chen Z, et al. System analysis of gene mutations and clinical phenotype in Chinese patients with autosomal-dominant polycystic kidney disease. *Scientific Reports.* 2016;6(1).

10. Kinoshita M, Higashihara E, Kawano H, et al. Technical Evaluation: Identification of Pathogenic Mutations in PKD1 and PKD2 in Patients with Autosomal Dominant Polycystic Kidney Disease by Next-Generation Sequencing and Use of a Comprehensive New Classification System. *PLoS One.* 2016;11(11):e0166288.

11. Mallawaarachchi AC, Hort Y, Cowley MJ, et al. Whole-genome sequencing overcomes pseudogene homology to diagnose autosomal dominant polycystic kidney disease. *Eur J Hum Genet.* 2016;24(11):1584-1590.

12. Raj S, Singh RG, Das P. Mutational screening of PKD2 gene in the north Indian polycystic kidney disease patients revealed 28 genetic variations. *Journal of Genetics.* 2017;96(6):885-893.

13. Sha Y-K, Sha Y-W, Mei L-B, et al. Use of targeted sequence capture and high-throughput sequencing identifies a novel PKD1 mutation involved in adult polycystic kidney disease. *Gene.* 2017;634:1-4.

14. Bullich G, Domingo-Gallego A, Vargas I, et al. A kidney-disease gene panel allows a comprehensive genetic diagnosis of cystic and glomerular inherited kidney diseases. *Kidney Int.* 2018;94(2):363-371.

15. Ranjzad F, Aghdami N, Tara A, Mohseni M, Moghadasali R, Basiri A. Identification of Three Novel Frameshift Mutations in the PKD1 Gene in Iranian Families with Autosomal Dominant Polycystic Kidney Disease Using Efficient Targeted Next-Generation Sequencing. *Kidney Blood Press Res.* 2018;43(2):471-478.

16. Abdelwahed M, Hilbert P, Ahmed A, et al. Mutational analysis in patients with Autosomal Dominant Polycystic Kidney Disease (ADPKD): Identification of five mutations in the PKD1 gene. *Gene.* 2018;671:28-35.

17. Xu D, Ma Y, Gu X, et al. Novel Mutations in the PKD1 and PKD2 Genes of Chinese Patients with Autosomal Dominant Polycystic Kidney Disease. *Kidney Blood Press Res.* 2018;43(2):297-309.

18. Kim H, Park HC, Ryu H, et al. Genetic Characteristics of Korean Patients with Autosomal Dominant Polycystic Kidney Disease by Targeted Exome Sequencing. *Sci Rep.* 2019;9(1):16952.

19. Dong K, Miao H, Jia X, et al. Identification of a pathogenic mutation in a Chinese pedigree with polycystic kidney disease. *Mol Med Rep.* 2019;19(4):2671-2679.

20. Wang T, Li Q, Shang S, et al. Identifying gene mutations of Chinese patients with polycystic kidney disease through targeted next-generation sequencing technology. *Mol Genet Genomic Med.* 2019;7(6):e720.

21. Mochizuki T, Teraoka A, Akagawa H, et al. Mutation analyses by next-generation sequencing and multiplex ligation-dependent probe amplification in Japanese autosomal dominant polycystic kidney disease patients. *Clin Exp Nephrol.* 2019;23(8):1022-1030.

22. Mantovani V, Bin S, Graziano C, et al. Gene Panel Analysis in a Large Cohort of Patients With Autosomal Dominant Polycystic Kidney Disease Allows the Identification of 80 Potentially Causative Novel Variants and the Characterization of a Complex Genetic Architecture in a Subset of Families. *Front Genet.* 2020;11:464.

23. Nielsen ML, Lildballe DL, Rasmussen M, Bojesen A, Birn H, Sunde L. Clinical genetic diagnostics in Danish autosomal dominant polycystic kidney disease patients reveal possible founder variants. *Eur J Med Genet.* 2021;64(4):104183.

24. Mallawaarachchi AC, Lundie B, Hort Y, et al. Genomic diagnostics in polycystic kidney disease: an assessment of real-world use of whole-genome sequencing. *Eur J Hum Genet.* 2021;29(5):760-770.

25. Elliott MD, James LC, Simms EL, et al. Mainstreaming Genetic Testing for Adult Patients With Autosomal Dominant Polycystic Kidney Disease. *Can J Kidney Health Dis.* 2021;8:20543581211055001.

26. Kim H, Kim HH, Chang CL, Song SH, Kim N. Novel PKD1 Mutations in Patients with Autosomal Dominant Polycystic Kidney Disease. *Lab Med.* 2021;52(2):174-180.

27. Hanlu Wang SD, Jianhui Zhang, Yi Li, Yumian Gan, Tao Lu, Yaobin Zhu4 Ning Lin, FT, Jiewei Luo. Analysis of mutations in six Chinese families with autosomal dominant polycystic kidney disease. *American journal of translational research.* 2022.

28. Abdelwahed M, Hilbert P, Ahmed A, et al. Autosomal dominant polycystic kidney disease (ADPKD) in Tunisia: From molecular genetics to the development of prognostic tools. *Gene.* 2022;817:146174.

29. Alzahrani OR, Alatwi HE, Alharbi AA, et al. Identification and Characterization of Novel Mutations in Chronic Kidney Disease (CKD) and Autosomal Dominant Polycystic Kidney Disease (ADPKD) in Saudi Subjects by Whole-Exome Sequencing. *Medicina (Kaunas).* 2022;58(11).

30. Suzuki Y, Katayama K, Saiki R, et al. Mutation Analysis of Autosomal-Dominant Polycystic Kidney Disease Patients. *Genes (Basel).* 2023;14(2).
